# The LUMIERE dataset: Longitudinal Glioblastoma MRI with expert RANO evaluation

**DOI:** 10.1038/s41597-022-01881-7

**Published:** 2022-12-15

**Authors:** Yannick Suter, Urspeter Knecht, Waldo Valenzuela, Michelle Notter, Ekkehard Hewer, Philippe Schucht, Roland Wiest, Mauricio Reyes

**Affiliations:** 1 ARTORG Center for Biomedical Engineering Research, University of Bern, Bern, Switzerland; 2 Radiology Department, Spital Emmental, Burgdorf, Switzerland; 3 Support Center for Advanced Neuroimaging, Inselspital, Bern, Switzerland; 4 Cantonal Hospital of Graubünden, Chur, 7000 Switzerland; 5 Institute of Pathology, Lausanne University Hospital and University of Lausanne, Lausanne, Switzerland; 6 Department of Neurosurgery, Inselspital, Bern, Switzerland

**Keywords:** Cancer imaging, CNS cancer, Magnetic resonance imaging

## Abstract

Publicly available Glioblastoma (GBM) datasets predominantly include pre-operative Magnetic Resonance Imaging (MRI) or contain few follow-up images for each patient. Access to fully longitudinal datasets is critical to advance the refinement of treatment response assessment. We release a single-center longitudinal GBM MRI dataset with expert ratings of selected follow-up studies according to the response assessment in neuro-oncology criteria (RANO). The expert rating includes details about the rationale of the ratings. For a subset of patients, we provide pathology information regarding methylation of the O^6^-methylguanine-DNA methyltransferase (MGMT) promoter status and isocitrate dehydrogenase 1 (IDH1), as well as the overall survival time. The data includes T1-weighted pre- and post-contrast, T2-weighted, and fluid-attenuated inversion recovery (FLAIR) MRI. Segmentations from state-of-the-art automated segmentation tools, as well as radiomic features, complement the data. Possible applications of this dataset are radiomics research, the development and validation of automated segmentation methods, and studies on response assessment. This collection includes MRI data of 91 GBM patients with a total of 638 study dates and 2487 images.

## Background & Summary

Glioblastoma (GBM) is a highly infiltrative brain tumor. To this day, no curative treatment for GBM patients is available. The current standard-of-care involves maximum safe surgical resection, radiotherapy, chemotherapy, and palliative treatment^1,2^. Its fast growth makes close disease monitoring paramount. The treatment response is routinely evaluated on Magnetic Resonance Imaging (MRI), according to the response assessment in neuro-oncology criteria (RANO)^3^. A quantitative part of RANO is concerned with measuring the active tumor appearing on contrast-enhanced T1-weighted MRI, a qualitative evaluation is based on abnormalities on the T2-weighted and fluid-attenuated inversion recovery (FLAIR) MRI.

The success of Machine Learning (including Deep Learning, Artificial Intelligence) has fueled research on automated tumor segmentation tools. Such tools show promising results on pre-operative imaging data^4,5^, and encouraging results have been presented for post-operative data and for assisting clinicians. The use of automated measurement tools based on automated segmentation methods was investigated (e.g.^6–8^).

The availability of annotated data — either performed manually or by an automated system — has empowered researchers to investigate sub-visual cues and complex image-based biomarkers in the field of radiomics^9^. For GBM, radiomics has been applied to outcome-related tasks such as overall survival prediction^5^, radiogenomics^10–12^, to detect pseudo-progression^13^, and progression based on MRI^14^.

Publicly accessible data is the cornerstone of research on Deep Learning for applications in Radiology. Data availability is critical to establishing new techniques and testing and benchmarking systems outside of the centers an algorithm is developed. Scientific journals increasingly emphasize the importance of external validation data and best practices in validation of new developments^15–17^. We aim to provide researchers with a first publicly available expert RANO rating on a longitudinal data set with MRI data, automated tumor segmentations, and a rich set of complementary information.

**Fig. 1:**
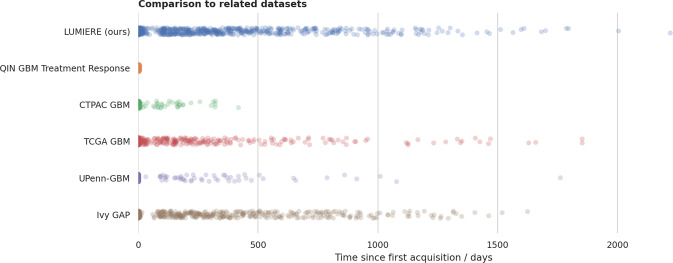
Comparison of similar GBM datasets regarding scan distribution on a time scale. Every circle corresponds to one study date. Only time points with known time since the first acquisition were considered for this figure.

We briefly describe publicly available datasets of MRI data from GBM patients below, and Fig. 1 shows the longitudinal distribution of acquisitions.

The **TCGA-GBM** dataset offers computed tomography (CT) and MRI data of 262 GBM patients. For 259 patients, MRI data with a total of 575 acquisition dates are available, stemming from eight different centers, and are predominantly acquired pre-operatively. Due to its link to The Cancer Genome Atlas (TCGA), clinical, genetic, and pathological information is available. 51% of the available images were acquired pre-operatively. Bakas *et al*. added segmentation labels and radiomics features to the pre-operative MRI for this dataset and made it publicly available^18,19^.

The **CPTAC-GBM** collection contains MRI data from 62 patients, with 140 acquisition dates. The imaging data with a follow-up period beyond three months is available for eight of these patients. The data is complemented with tissue slice images.

The **IvyGap** dataset offers data of 39 patients, and is complemented by *in situ* hybridization (ISH) and RNA sequencing information. For 37 of these patients, the collections also includes post-operative MRI^20^.

The **QIN GBM Treatment Response** dataset^21,22^ contains MRI data from 54 patients with a total of 105 acquisition time points. The post-operative MRI scans were performed after a maximum of eight days after the pre-operative study.

The recently published **UPenn-GBM** dataset^23^ contains MRI data from 630 patients and includes 611 pre- and 60 post-operative scans. The data further includes diffusion tensor imaging (DTI), dynamic susceptibility contrast (DSC) imaging, pathology data, and information regarding the age, sex, extent of resection and survival time.

In the 2021 edition, the **Brain Tumor Segmentation (BraTS)** challenge offered in its training set pre-operative MRI data of 1251 brain tumor patients with tumor segmentations. Four MRI sequences are provided: pre- and post-contrast T1-weighted (T1, CT1), T2-weighted (T2), and fluid-attenuated inversion recovery (FLAIR). The tumor segmentation includes the contrast-enhancement, necrotic region, and edema with potentially non-enhancing tumor. It contains pre-operative data from the TCGA-GBM, TCGA-LGG, IvyGAP, and CPTAC-GBM collections^5,24^.

The prior work that was based on this or subsets of this dataset is focused on automated tumor burden quantification. Meier *et al*.^25^ investigated the capability of automated segmentation tools for automated longitudinal tumor size measurements. Keller-Weldon *et al*.^26^ and Porz *et al*.^27^ compared automated and manual bi-dimensional tumor measurements. Meier *et al*.^28^ investigated the automated quantification of the extent-of-resection. Suter *et al*.^29,30^ used the pre-operative data to test the robustness of radiomic features and machine learning classifiers in the context of multi-center studies.

In this dataset, we release the longitudinal MRI data of 91 GBM patients who underwent pre-operative MRI between August 2008 and December 2013, with a follow-up period until 2017. The anonymized imaging data is complemented by expert ratings according to the RANO guidelines, patient age at time of diagnosis, sex, and overall survival time. The expert ratings, which we consider a key contribution of this dataset, include the disease state (progressive disease, stable disease, partial response, or complete response), the rating rationale, and bi-dimensional Macdonald^31^ measurements for lesions above the measurability threshold. The output of two state-of-the-art automated segmentation tools DeepBraTumIA (https://www.nitrc.org/projects/deepbratumia), and HD-GLIO-AUTO^6,32,33^, are included, both based on the highly successful U-Net deep learning architecture^34^. Furthermore, we include all radiomic features from the PyRadiomics package^35^ for each segmentation label, as well as features describing the Co-occurrence of Local Anisotropic Gradient Orientations (CoLlAGe) features^36^. The imaging information is accompanied by the MRI acquisition parameters for each image to enable researchers to correct for confounding factors and investigate the impact of imaging differences, e.g., on radiomic features and machine learning algorithms. The provided pathology information includes the O^6^-methylguanine-DNA methyltransferase promoter methylation (MGMT) and isocitrate dehydrogenase 1 (IDH1).

## Methods

The records of 91 GBM patients who underwent pre-operative MRI between 2008 and 2013 were reviewed retrospectively, building on the study population of Schucht *et al*.^37^ based on the WHO 2016 classification scheme^38^. All patients were treated with surgical resection and temozolomide-based chemo-radiation at the Inselspital, the University Hospital of Bern, Switzerland. Two patients received Avastin as a second-line treatment, which is noted in the clinical information provided in the dataset. The cantonal ethics committee of Bern approved the studies and waived written informed consent.

Table 1 gives an overview of the patients demographics. The mean survival was at 589 days (19 months) with a standard deviation of 334 days. The patient’s mean age at the time of the first resection was 62.4 years, with a standard deviation of 10.3 years. Derived from the expert RANO ratings, we derived the time to progression as the time span from the first resection to the first follow-up rated as progressive disease.

**Table 1:** Patient demographics of the single-center dataset.

	Mean	Standard deviation	Minimum	Maximum
Overall Survival/days	589.1	334.0	25	1412
Time to progression/days	305.4	266.1	25	1537
Age at resection/years	62.4	10.3	39.7	80.4
Sex	female: n = 44, male: n = 47			
MGMT methylation	methylated: n = 37, unmethylated: n = 43, not available: n = 11			
IDH	wild-type: n = 57, negative* n = 10, mutant: n = 1, not available: n = 23			

MGMT: O^6^-methylguanine-DNA methyl-transferase promoter methylation, IDH: isocitrate dehydrogenase, *: IDH1 negative by immunohistochemistry, sequencing would be required for a conclusive result. In case no (radiological) progression was noted, the time to progression was set to equal the overall survival time.

The study population included 44 women and 47 men. MGMT methylation was found in 37 patients, 43 patients with unmethylated MGMT, and the MGMT status was unavailable for 11 patients. IDH1 was determined as wild-type for 57 patients, 1 patient was IDH-mutant, and IDH information was unavailable for 23 patients. IDH immunohistochemistry was negative for 10 patients, where sequencing would have been necessary for a definite result.

### Expert RANO rating

The whole dataset was retrospectively reviewed and evaluated according to the RANO guidelines by an expert neuroradiologist with 14 years of experience specializing in neuro-oncological diagnostics (U.K.). We note that in some cases, the rating is present even though not all four MRI sequences are available since a rating is possible with a subset of the images. The dataset contains 616 expert-rated time points.

### Anonymization

All imaging data were skull-stripped to prevent identification by a patient’s skull shape. The skull-stripping was performed with HD-BET^32^. Both segmentation tools were used on the non-skull-stripped images since both include a brain extraction step. We only retained outputs for both segmentation tools after skull-stripping and renamed the files produced to not contain identifying information. We exclusively provide images in the NIfTI file format to avoid leakage of sensitive metadata from DICOM headers. All dates and time spans were set to be relative to the pre-operative image acquisition. The relative time-spans were subsequently converted to week counts to blur the precise follow-up intervals while preserving enough precision to retain the value of the temporal information. If two study dates fall into the same week, the relative ordering is indicated with a further number, most often the case for pre- and post-operative imaging around surgical resections (e.g., week-000-1 and week-000-2). A follow-up marked at week four may have been acquired between 28 and 35 days after pre-operative acquisition. The patient numbering was created randomly.

### MRI data

**Fig. 2:**
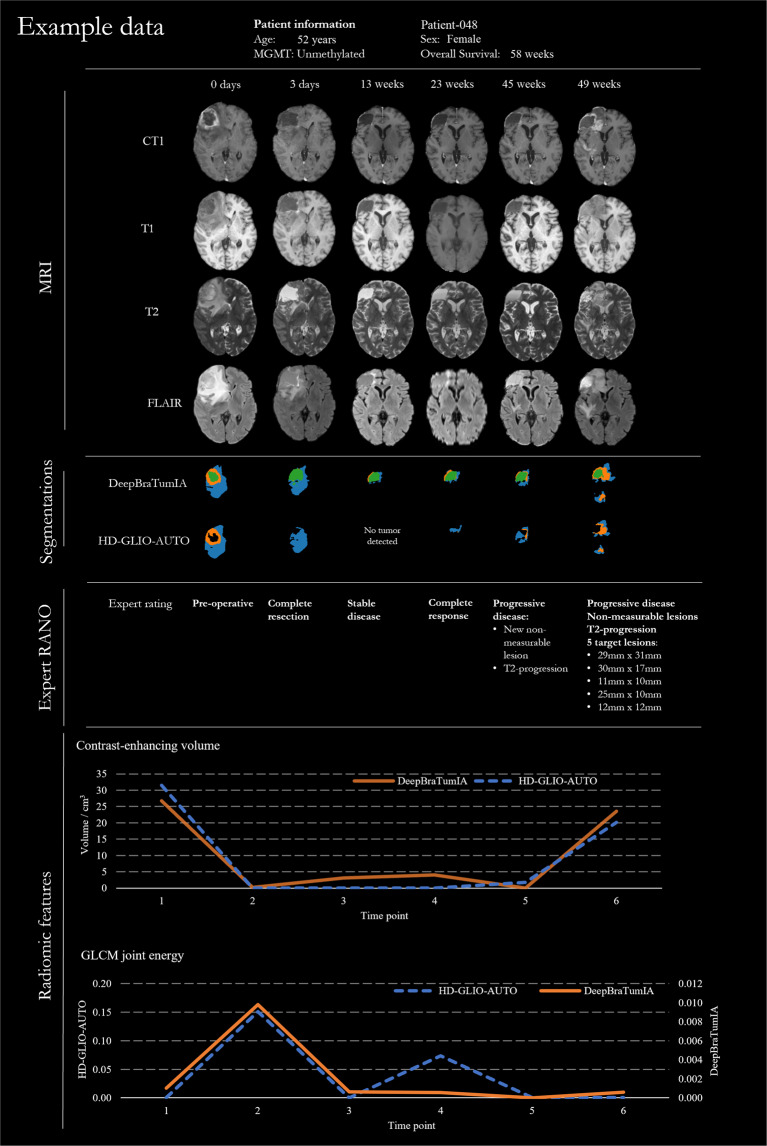
Example of longitudinal GBM data. CT1/T1: T1-weighted post-/pre-contrast, T2-weighted, and fluid-attenuated inversion recovery (FLAIR). For this patient, the whole contrast enhancement was completely resected. After a phase of complete response, progression occurred with a very fast growth rate.

A summary of the MRI acquisition parameters for the provided MRI sequences is listed in Table 2, and an example series of longitudinal images is shown in Fig. 2. For each MRI image in the dataset, we provide details of the acquisition parameters in a CSV file, such as the vendor, model, timing, voxel size, slice spacing, field strength, flip angle, and the specific absorption rate. 95% of the 2487 provided MRI images have been acquired on Siemens scanners, 3% on Philips scanners (Philips Medical Systems/Philips Healthcare), and 2% on scanners from GE Medical Systems.

**Table 2:** Acquisition parameters retrieved from the DICOM information.

	CT1	T1	T2	FLAIR
Field strength/T	1.94 ± 0.69	1.92 ± 0.68	1.92 ± 0.68	1.91 ± 0.67
Rows	284.52 ± 80.45	348.66 ± 90.78	388.7 ± 119.38	415.48 ± 112.83
Columns	281.67 ± 73	317.87 ± 77	362.84 ± 101	358.53 ± 105
Slices	156.75 ± 13.28	148.66 ± 11.56	153.50 ± 13.30	155.91 ± 13.90
Thickness/mm	1.25 ± 0.96	3.77 ± 1.48	2.95 ± 1.98	3.73 ± 0.68
Slice spacing/mm	0.39 ± 2.25	4.15 ± 2.25	2.95 ± 2.25	4.39 ± 0.65
In-plane voxel size/mm	0.94 ± 0.17	0.71 ± 0.21	0.70 ± 0.26	0.60 ± 0.21
Echo time/ms	4.85 ± 3.31	6.02 ± 5.98	237.72 ± 149.02	94.59 ± 34.08
Repetition time/ms	1767 ± 578	782 ± 619	3761 ± 891	8376 ± 780
Inversion time/ms	1026 ± 119	977 ± 173	—	2441 ± 147
Flip angle/°	17.69 ± 18.39	62.76 ± 37.43	130.77 ± 18.85	150.8 ± 18.87
SAR	0.06 ± 0.14	0.34 ± 0.31	0.30 ± 0.30	0.29 ± 0.24

All values are shown as mean ± standard deviation. SAR: Specific absorption rate, CT1: T1-weighted post-contrast, T1: T1-weighted pre-contrast, T2: T2-weighted, FLAIR: Fluid attenuated inversion recovery. The voxel size is indicated as a single value since all voxels were quadratic in plane.

The skull-stripped, unregistered images are provided to enable further studies not confounded by potential resampling artifacts.

### Automated segmentation

Automated tumor segmentations were created with DeepBraTumIA and HD-GLIO-AUTO. Both methods include a co-registration of the four MRI sequences. HD-GLIO-AUTO registers all sequences to a reference image chosen automatically during processing, and DeepBraTumIA registers to an atlas such that all studies may be analyzed in the same space. HD-GLIO-AUTO was slightly modified to retain the transformation used for co-registration of the MRI sequences to enable researchers to perform analysis in the original image space. DeepBraTumIA also provides the possibility of studying the segmentation in the original patient space. Hence we provide the back-transformed segmentations to the original image sequence for both segmentation methods.

HD-GLIO-AUTO provides segmentations for the contrast-enhancing tumor and the T2-signal abnormality. DeepBraTumIA outputs labels for necrosis, contrast enhancement, and edema.

We release automated segmentation of both tools to provide a richer algorithm benchmark and enable researchers to assess the impact of segmentation variability on different parameters of interest (e.g., radiomic biomarkers). The automated segmentation tools require all four MRI sequences as input, which is the case for 599 study dates.

### Radiomic features

Radiomic features from the PyRadiomics package (version 3.0.1) are extracted on the co-registered and resampled images for each segmentation label. Feature types include first-order statistics, 3D shape, gray level co-occurrence matrix (GLCM), gray level run length matrix (GLRLM), gray level size zone matrix (GLSZM), neighboring gray-tone difference matrix (NGTDM), and gray level dependence matrix (GLDM) features. For extraction, z-score normalization, scaling by a factor of 100, and intensity shifting by 300 were applied to harmonize the value range and ensure that the majority of voxels have a positive intensity value. The bin width was set to 5.

The CoLlAGe features were calculated with a singular value decomposition radius of 5 and 64 unique angles. Since CoLlAGe features are calculated voxel-wise, we provide feature maps for the whole segmentation foreground area. For convenience to display, the feature vector images are split into their primary and secondary components. The CoLlAGe feature extractor requires a minimum of 50 image slices. For some cases, especially for HD-GLIO-AUTO, the reference image had a lower slice count. To provide a more complete feature set, we extracted this feature type for HD-GLIO-AUTO after resampling the images to 1 mm iso-voxels for all cases. The resampling was used for all cases (even for those with enough original slices) to provide a consistent processing across time points and patients. We provide the code used to generate the needed auxiliary steps and files in the linked GitHub repository. These two feature vector images are provided for all MRI sequences and both automated segmentation tools.

While these feature extraction settings may be used as a feasible baseline, we encourage researchers to customize the parameters for a given target task.

## Data Records

All records for this data collection are available through Figshare^39^. MRI images and tumor segmentations are stored in The Neuroimaging Informatics Technology Initiative (NIfTI) format, maintaining raw medical image coordinates. Clinical and pathology data, radiomics features, MRI acquisition information, as well as the expert rating are stored in comma-separated values (CSV) files. Furthermore, we provide CSV files detailing the data completeness for each study date and provide an extensive readme document elaborating on the origin of the image or how it was derived.

## Technical Validation

Each study was visually assessed to ensure skull-stripping was of sufficient quality to ensure patient anonymization. Segmentation outliers from both automated segmentation tools were intentionally left in the dataset to enable studies focusing on common failure modes of automated segmentation tools.

**Fig. 3:**
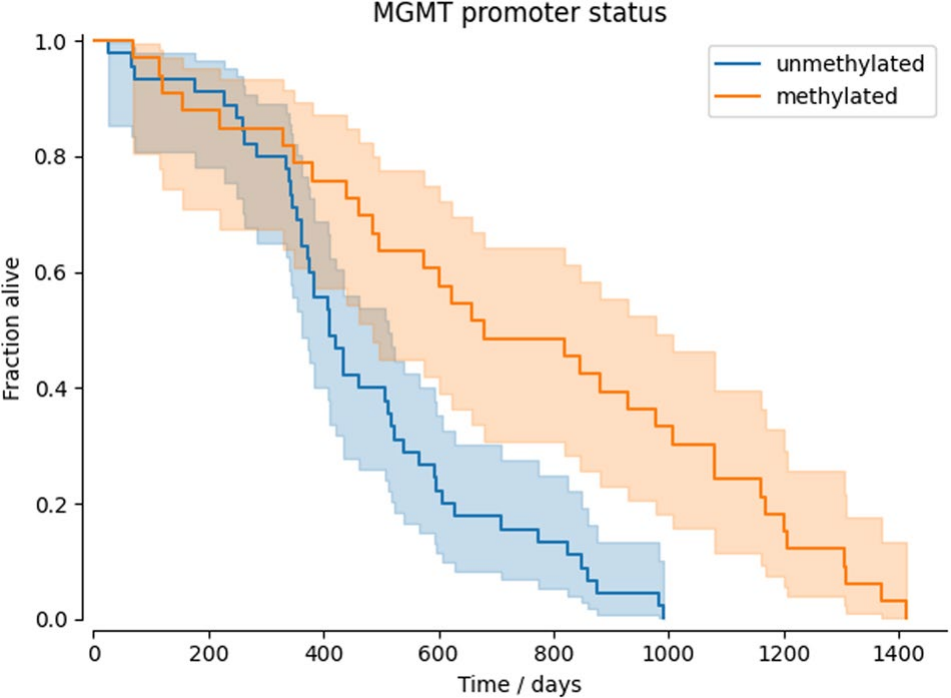
Kaplan-Meier (KM) curves for MGMT methylated and unmethylated groups. As expected, patients with MGMT methylation have a much higher median survival.

The pathology data was used to ensure our findings were in line with expected and prior clinical findings. Figure 3 shows the Kaplan-Meier curves for our patient cohort stratified by the MGMT promoter status. Consistent with the literature, we observe a much higher median survival time for patients with methylated MGMT status^40,41^.

**Fig. 4:**
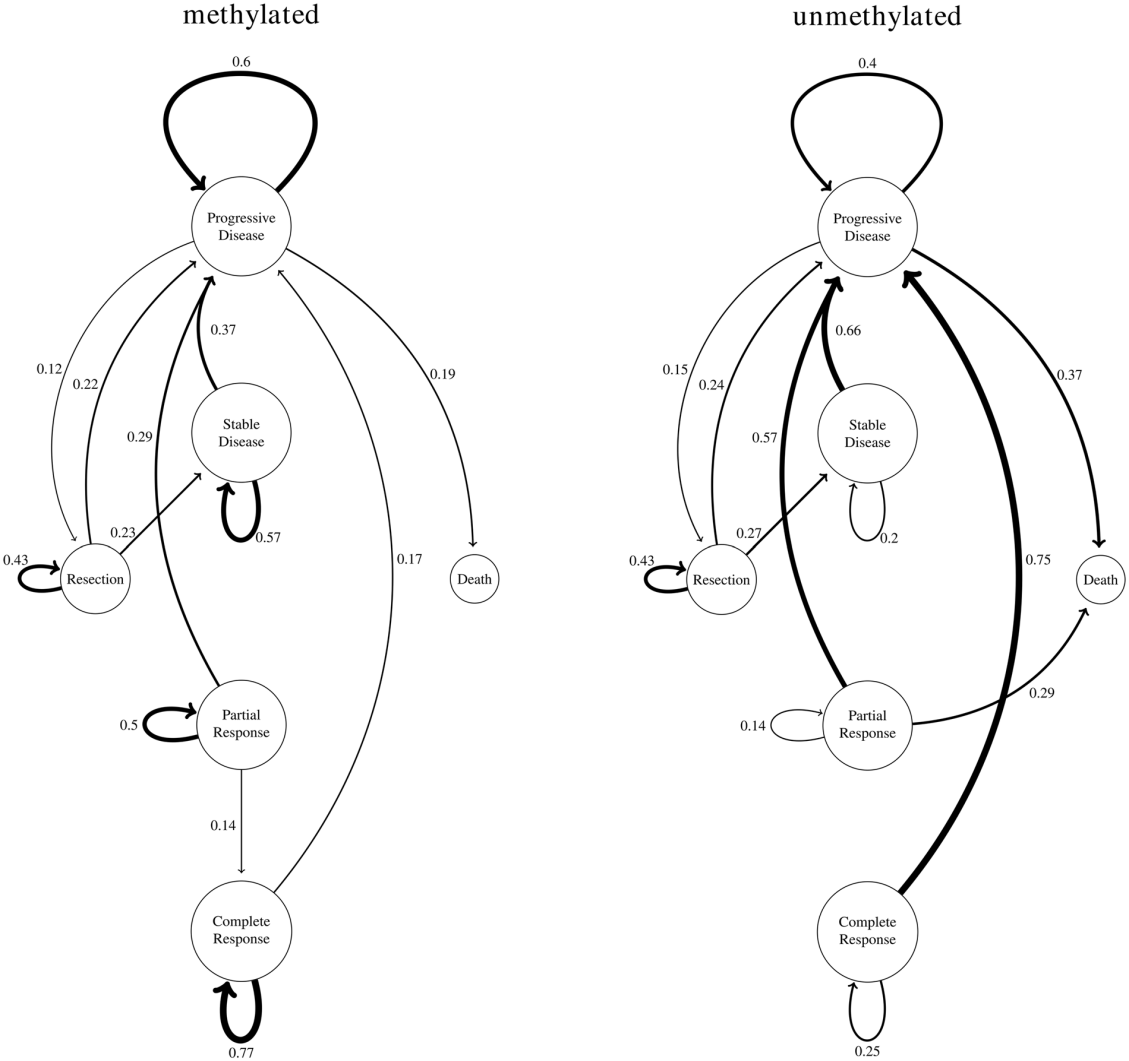
State transitions stratified by the MGMT promoter methylation status. Transitions with a probability of less than 0.1 are not shown for clarity. For MGMT promoter methylation, we observed about the same probability of going directly to progression after resection but more stable states than for the unmethylated status. For the unmethylated MGMT promoter status, the transition probabilities into the progressive disease state were much higher. We note that the sample size for staying in the complete response state was very small for both cases, and the large difference between the two methylation statuses may be considered with caution.

The expert rating was checked for unfeasible disease state transitions and subsequently revised if needed. We furthermore investigated the transition probability between disease states for patients with methylated and unmethylated MGMT promoter status. This was calculated by a simple occurrence count of state changes normalized by the total number of transitions from a given state. As shown in Fig. 4, we confirm the poorer prognosis of patients with unmethylated MGMT status on a longitudinal level with a lower probability of complete treatment response and higher probabilities for a fast disease progression.

## Usage Notes

To visually assess the imaging data, we recommend software such as 3D Slicer^42^, or ITK-Snap^43^. We provide example Python scripts for other researchers to get a fast overview of the imaging data, as well as to extract radiomic features with customized settings. This dataset may only be used for non-commercial purposes.

## Data Availability

The code used for processing this dataset is publicly available in our GitHub repository (https://github.com/ysuter/gbm-data-longitudinal). The Python and Bash scripts are available to reproduce and customize the extraction of radiomics features.
